# Harmonizing vaccine and infrastructure development to tame cholera outbreaks across Africa

**DOI:** 10.1038/s41467-024-49077-4

**Published:** 2024-06-01

**Authors:** Amira Mohamed Taha, Hussam Mahmoud, Emad M. Hassan, Mohamed M. Ghonaim

**Affiliations:** 1 Doctor, Faculty of Medicine, Fayoum University, Fayoum, Egypt; 2 George T. Abell Professor in Infrastructure, Department of Civil and Environmental Engineering, Colorado State University, Colorado, CO USA; 3 Reseach Scientist, Department of Civil and Environmental Engineering, Colorado State University, Colorado, CO USA; 4 Doctor, Faculty of Medicine Menoufia University, Menoufia, Egypt

**Keywords:** Infectious diseases, Health policy, Civil engineering, Public health

## Abstract

Vaccines and clean water shortages continue to give rise to cholera outbreaks in Africa. Coordinated efforts to increase vaccine distribution and improve physical infrastructure are needed while considering future outbreaks and water demands due to conflicts and climate events.

*Cholera*, caused by the Vibrio cholerae bacteria, spreads in areas without safe water and proper sanitation and can be fatal in hours if left untreated. Since 2023, 18 nations in the region have been dealing with one of the largest cholera outbreaks in years, with about 335,059 cases and 6,197 deaths, with a case fatality ratio of 1.8% reported as of March 3, 2024^1^. Six different countries (the Democratic Republic of the Congo, Zambia, Zimbabwe, Ethiopia, Mozambique, and the United Republic of Tanzania) are currently experiencing a crisis^2^. Specifically, the Democratic Republic of the Congo and Malawi are among the hardest hit, with the highest number of cases (78,107), and deaths (1,774 deaths), respectively^1^. The ongoing cholera epidemic in Eastern and Southern Africa further highlights the intricate interplay of factors that contribute to disease spread.

Drinking contaminated water and poor food preservation methods are the major contributors of cholera transmission. Other risk factors include bathing in the river, eating dried fish, not boiling drinking water, living with cases having acute diarrhea, traveling and eating outside the home, and consuming unrefrigerated leftover food. Additionally, underscoring the role of socio-economic characteristics, factors such as rapid urbanization, poor housing, and overcrowding in urban slums, are crucial for understanding possible causes of sustained cholera transmission in Africa. While vaccine distribution and improving water infrastructure are vital, effective prevention and control of cholera depend on robust water, sanitation, and hygiene (WASH) practices. These include ensuring access to clean water, promoting personal hygiene and handwashing, safe food handling, and the prompt detection and clinical management of cases. We address, as this epidemic highlight, the critical need for comprehensive and long-term cholera control strategies addressing the role of vaccines and infrastructure in the rise of cholera outbreaks in Africa.

## Key challenges in accessing cholera vaccines and safe water

An efficient two-dose oral cholera vaccination regimen exists, providing immunity for about three years, but a global vaccine scarcity has compounded the situation^3^. These shortages are not only the result of a global increase in cholera cases but also of vaccine producers’ collective lack of interest in producing cholera vaccines. The oral cholera vaccine is inexpensive and requires production at large sales to create profit, which suggests that the vaccine may be of little financial interest to most enterprises in high-income countries. The consequent vaccine scarcity prompted the International Coordinating Group (ICG) on Vaccine Provision to abandon the conventional two-dose cholera vaccine protocol in favor of a single dosage to assure greater coverage^4^. The reduced dose of the cholera vaccine is a temporary solution since results show identical efficacy in the first year and lesser effectiveness over the first four years after immunizations compared to normal vaccine doses^5^.

Gavi reported that 48 million vaccination doses were utilized in 2022 and 2023, 10 million more than the previous decade^6^. The vaccine shortage will be exacerbated by a manufacturing decision to discontinue production of the Shanchol vaccine, one of just two cholera vaccines accessible for large humanitarian efforts. WHO recommends cholera vaccination for emergency and relief workers likely to be exposed to cholera patients or contaminated food or water, as well as the special population, including HIV-infected individuals, Pregnant women, and populations in closed institutions^3^.

Similar to the shortage of vaccines, Africa has been going through a water crisis primarily due to water scarcity, lack of adequate water and wastewater infrastructure, and improper water management and governance^7^. Half of all countries on the continent scored very low on having basic water infrastructure^7^. Three of every five people on the continent do not have access to safe drinking water, and three of every four lack access to enough sanitation and hygiene services^8^. These conditions are further exacerbated by changing weather patterns, climate change, and phenomena such as El Niño leading to floods and droughts. The floods in late 2023 in eastern Africa destroyed thousands of homes, and nearly two million people were forced to relocate to temporary shelters with crowded and unsanitary living conditions, which may increase the spread of disease^9^.

Another major obstacle in confronting such outbreaks is armed conflicts. Conflicts exhaust response capacities, healthcare infrastructure, and material and financial ability to provide sufficient treatment and prophylactic measures. In the Democratic Republic of the Congo, a key epicenter of the current outbreak, cases are concentrated in the conflict-torn east, where entire communities have been razed to the ground, and floods and landslides have occurred^10^. Years of ongoing multidimensional violence have resulted in huge population displacement (up to 7 million)^11^, reducing Congolese citizens’ resilience to such outbreaks. When compounded with natural hazards and extreme events, large and long-lasting epidemics present extra challenges for healthcare systems, including damage to physical infrastructure, shortage in supplies, and reduced availability of personnel, who are already overburdened by responding to various diseases in challenging conditions^10,12^.

## Infrastructure improvement and vaccine access recommendations

The Global Task Force on Cholera Control has set goals to cut cholera mortality by 90% and eradicate cholera in up to 20 countries by 2030. These targets necessitate investments in safe water and sanitation infrastructure for all. Capacity must also be built for preventative, readiness, and preparedness operations, such as simplifying the supply chain for critical diagnostics. Vaccine manufacturing must be increased globally and in severely afflicted areas, including many regions in Africa. Even though Ghana has demonstrated that it is possible to decentralize and increase sustainable vaccine production in Africa, becoming the continent’s first manufacturer of a cholera vaccine^13^, the continent provides less than 1% of the vaccines it uses.

While vaccine production is important in reducing the spread of cholera, the lack of basic water infrastructure to produce safe and clean drinking water and the continued struggle to achieve basic hygiene services make any progress toward vaccine development rather irrelevant. In 2022, UNICEF reported that a 12-fold increase in current rates of progress on safely managed drinking water, a 20-fold increase for safely managed sanitation, and a 42-fold increase for basic hygiene services are needed to achieve the Sustainable Development Goal targets in Africa^14^. Therefore, substantial investments are needed to provide clean water, basic sanitation, and hygiene. While water governance and management are key considerations, developing basic water infrastructure is a prerequisite to ensure accessibility to clean drinking water.

Partnerships and capital investment are urgently required throughout African countries to tackle vaccination and clean water shortages. Integrating vaccine distribution with water infrastructure development is critical for cholera prevention. Priority should be given to analyzing and correcting water infrastructure inadequacies in areas with high cholera rates and inadequate vaccination access. It is critical to establish community-run water purification centers that can also serve as vaccination locations. Climate change and population expansion must be taken into account while addressing water constraints. Agreements that encourage water diplomacy and resource sharing across the continent are critical. Furthermore, it is vital to take care of the entire water supply chain, from source to consumption for effective management including frequent quality assessments of water sources. Water must be transported in clean, covered containers to avoid exposure to pollutants, stored in sanitized containers at home and, ideally, treated before use to eradicate microorganisms, such as boiling or chlorinating. It is also critical to educate populations about the dangers of unclean drinking water and good hygiene procedures. Regular community participation and hygiene promotion can greatly raise awareness and adherence to clean water practices, lowering the risk of cholera and other waterborne illnesses.

**Fig. 1:**
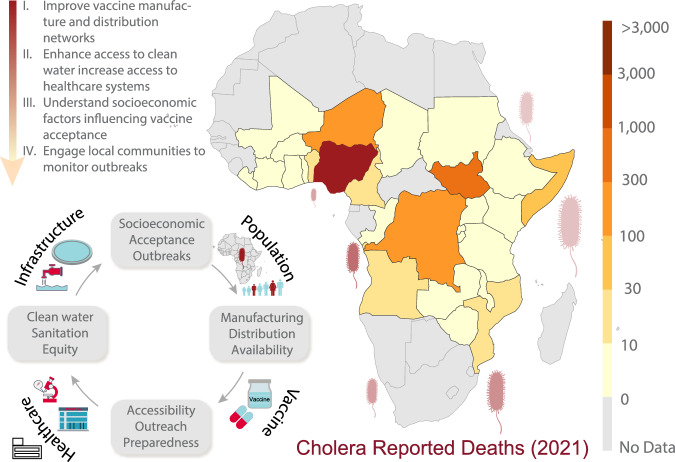
Illustration of the required coordinated efforts to increase vaccine distribution and improve physical infrastructure to reduce cholera outbreaks, along with data showing reported deaths from cholera outbreaks across Africa in 2021 (Data Source: Outworld in Data^15^).

Improving access to medical services and vaccine delivery, particularly in remote regions, as well as expanding vaccine networks, can help patients receive treatments and vaccines promptly. Mapping healthcare and water access against disease data can help identify important needs and predict the impact of climate change and conflicts. Improving logistics  by boosting storage at vaccine sites and introducing mobile units with water purification while lowering their exposure to natural and man-made hazards is critical. Integrating Cholera vaccinations into health systems, as well as enhancing outreach and collaborations, can help to increase vaccine uptake and disease monitoring. Health systems in high-risk areas should create emergency plans to deal with anticipated patient surges. Finally, effective epidemic control and the protection of vulnerable populations require close collaboration among nongovernmental organizations (NGOs), local authorities, communities, governments, and professionals (Fig. 1).

## Conclusion

Cholera epidemics and inadequate infrastructure in Africa necessitate urgent integrated policies that improve water and sanitation systems and encourage local vaccine manufacture and delivery, such as Ghana’s initiative. Improving vaccine distribution, particularly in rural regions, and incorporating it into healthcare systems is critical. Mapping existing water, health, and transportation infrastructure and predicting future infrastructure needs as influenced by climate change and armed conflicts is also important. In addition, community-based collaborations serve as essential for effective disease prevention and treatment.

## References

[1] World Health Organization. Cholera in the WHO African Region. https://iris.who.int/bitstream/handle/10665/376303/AFRO%20Cholera%20Bulletin.54.pdf (2024).

[2] World Health Organization. *Global strategic preparedness, readiness and response plan for cholera*. https://www.who.int/publications/m/item/global-strategic-preparedness-readiness-and-response-plan-for-cholera (2023)

[3] World Health Organization. (2018). Cholera vaccine: WHO position paper, August 2017—Recommendations. Vaccine.

[4] World Health Organization (WHO). Shortage of cholera vaccines leads to temporary suspension of two-dose strategy, as cases rise worldwide. https://www.who.int/news/item/19-10-2022-shortage-of-cholera-vaccines-leads-to-temporary-suspension-of-two-dose-strategy-as-cases-rise-worldwide (2022).

[5] Franke MF (2018). Long-term effectiveness of one and two doses of a killed, bivalent, whole-cell oral cholera vaccine in Haiti: an extended case-control study. Lancet Glob. Heal..

[6] World Health Organization (WHO). *Gavi Alliance MS Roadmap for Oral Cholera Vaccines 4: The Market Shaping Goal Ensure healthy markets for vaccines and related products*. https://reliefweb.int/report/world/gavi-alliance-ms-roadmap-oral-cholera-vaccines-4-market-shaping-goal-ensure-healthy-markets-vaccines-and-related-products (2023).

[7] Oluwasanya, G., Perera, D., Qadir, M. & Smakhtin, V. *Water Security in Africa: A Preliminary Assessment*. **13**, 46 (2022).

[8] UNICEF and WHO. *Progress on Drinking Water, Sanitation and Hygiene in Africa 2015-2020: Five Years into the SDGs*. https://data.unicef.org/resources/progress-on-drinking-water-sanitation-and-hygiene-in-africa/ (2022).

[9] Oxfam. East Africa’s floods decimate almost entire season harvest and leave over four million people with no food or income. https://reliefweb.int/report/ethiopia/east-africas-floods-decimate-almost-entire-season-harvest-and-leave-over-four-million-people-no-food-or-income (2023).

[10] World Health Organization (WHO). A new resolve to eliminate cholera in DRC. https://www.who.int/news-room/feature-stories/detail/a-new-resolve-to-eliminate-cholera-in-drc (2023).

[11] Betts A, Omata N, Siu J, Sterck O (2023). Refugee mobilities in East Africa: understanding secondary movements. J. Ethn. Migr. Stud..

[12] Hassan EM, Mahmoud HN (2021). Orchestrating performance of healthcare networks subjected to the compound events of natural disasters and pandemic. Nat. Commun..

[13] Adams, C. N. Africa: Ghana Manufactures First Cholera Vaccine in Africa. https://medaditus.org/news-articles/africa-ghana-manufactures-first-cholera-vaccine-in-africa/ (2023).

[14] UNICEF. Africa to drastically accelerate progress on water, sanitation and hygiene. (2022).

[15] Ourworldindata. Cholera reported deaths, 2021. https://ourworldindata.org/grapher/number-of-reported-cholera-deaths (2022).

